# Broadband perfect Littrow diffraction metasurface under large-angle incidence

**DOI:** 10.1515/nanoph-2024-0622

**Published:** 2025-02-10

**Authors:** Jingyuan Zhu, Siliang Zhou, Tao He, Chao Feng, Zhanshan Wang, Siyu Dong, Xinbin Cheng

**Affiliations:** Institute of Precision Optical Engineering, School of Physics Science and Engineering, Tongji University, Shanghai 200092, China; MOE Key Laboratory of Advanced Micro-Structured Materials, Shanghai 200092, China; Shanghai Frontiers Science Center of Digital Optics, Shanghai 200092, China; Shanghai Professional Technical Service Platform for Full-Spectrum and High-Performance Optical Thin Film Devices and Applications, Shanghai 200092, China; Shanghai Institute of Intelligent Science and Technology, Tongji University, Shanghai 200092, China

**Keywords:** large-angle, Littrow-mounting, non-local metasurface, broadband diffraction

## Abstract

Littrow diffraction devices are commonly used in the laser field (e.g., laser resonators and spectrometers), where system integration requires larger incidence angles and perfect broadband efficiency. Compared to traditional diffraction devices, which struggle to manipulate light paths under large-angle incidence, metasurfaces has the potential to enhance the broadband efficiency. Despite quasi three-dimensional metasurfaces effects, only perfect anomalous reflection under normal incidence at limited wavelengths was achieved due to energy flow mismatch in the broadband Littrow configuration. Here, we propose a supercell metasurface capable of regulating broadband non-local responses. The metasurface effectively suppresses non-local responses under Littrow mounting, while providing sufficient non-local responses through strong structural coupling effects when the incidence deviates from the Littrow mounting. A large-angle broadband Littrow diffraction metasurface in the mid-infrared spectrum (3.11 µm ∼ 3.52 µm) has been successfully realized, with 99 % efficiency at Littrow angle of 70°. Our results break through the bandwidth limitations of perfect diffraction, providing robust support for the practical applications of metasurfaces in Littrow diffraction devices.

## 1 Introduction

Littrow diffraction is a special phenomenon in which the first-order diffraction angle is equal to the incidence angle, aimed at achieving the highest efficiency [1]. It is widely used in the laser field, including in optical communication [2], imaging systems [3], [4], ultrathin lenses [5], [6], [7], beam combining [8], [9], [10], [11], [12] and spectral analysis [13], [14]. With the advancement of optical systems, diffraction devices require larger Littrow angles to meet the growing demands for integration, as well as broadband efficiency approaching 100 % to enable more complex functionalities. Currently, the most commonly used Littrow diffraction devices are metallic blazed gratings [15] and multilayer film gratings [16], [17], [18]. By designing the structure, these gratings can achieve high-efficiency diffraction at a specific Littrow angle, which is also known as the blaze angle. Theoretically, the absorption loss of metallic materials limits the efficiency of blazed gratings, while the reflective properties of multilayer films restrict the bandwidth [19]. Furthermore, larger incidence angles require even higher line densities, which make the fabrication of traditional gratings increasingly challenging. As a result, achieving broadband perfect Littrow diffraction under large-angle incidence is a significant challenge for traditional diffraction devices.

In recent decades, the introduction of metamaterials and metasurfaces has enabled more powerful control over optical fields. They allow for flexible control of the amplitude, phase and polarization of electromagnetic waves [20], [21], [22], [23], [24]. Alù proposed the use of periodic arrays of carefully tailored bianisotropic inclusions to achieve perfect wavefront transformation, which is called metagrating [25]. Based on coupled Bloch mode theory, Dong used multilayer freeform metagratings to achieve depolarized perfect Littrow diffraction [26]. However, the Bloch modes become more complex at larger incidence angles, making it challenging to be manipulated by metagratings.

Generally, when designing/simulating metasurfaces, the interactions between adjacent structures are often ignored, which are referred to as local metasurfaces. In contrast, non-local metasurfaces manipulate the spectrum by utilizing modes supported by many adjacent unit cells. Compared to metagratings, non-local metasurfaces can achieve stronger gain responses, enabling anomalous reflection of incident light at larger angles. Most metasurfaces exhibit the necessary effective non-local response through surface waves or evanescent waves, producing effects similar to gain and loss [27], [28], [29], [30]. Nevertheless, these metasurfaces still struggle to achieve 100 % reflectivity due to the metallic materials with high absorption losses at optical frequencies. It wasn’t until the introduction of all-dielectric quasi-three-dimensional subwavelength structures that perfect anomalous reflection in the optical frequency range was successfully achieved. Based on energy flow manipulation theory, He designed multilayer films with tailored thicknesses to alter the phase response at specific wavelengths, thereby achieving effective non-local control of light [31]. However, the existing energy flow theory was designed for normal incidence, and under Littrow incidence, the multilayer films provide almost no control. Additionally, as the wavelength deviates from the design wavelength, the non-local response required by the metasurface also changes, which means that independent control at a specific wavelength is insufficient to meet the demands of a broader bandwidth. The current metasurfaces face significant challenges in achieving broadband perfect diffraction under Littrow incidence due to the limitations in multilayer film design.

Here, we propose a novel Littrow diffraction metasurface that addresses energy flow mismatches under large angle incidence through non-local responses, comprising supercell structures and aperiodic multilayer films. Since achieving appropriate energy flow at specific locations is a necessary condition for perfect diffraction, the designed metasurface adjusts the broadband non-local response to ensure that the energy flow meets the requirements at different wavelengths. The supercell structures break the periodicity of simple metasurfaces, enhancing the lateral energy flow transfer through strong non-local effects. Simultaneously, the aperiodic multilayer films offer flexible control to ensure the energy flow meets the needs for perfect diffraction. We designed a perfect Littrow diffraction metasurface for the mid-infrared band by scanning the parameters of the supercell structures and optimizing the thickness of the multilayer films. As a proof of concept, we achieved a broadband perfect Littrow diffraction in the range of 3.11 µm ∼ 3.52 µm, with 99 % efficiency at Littrow angle of 70°.

## 2 Theory

**Figure 1:**
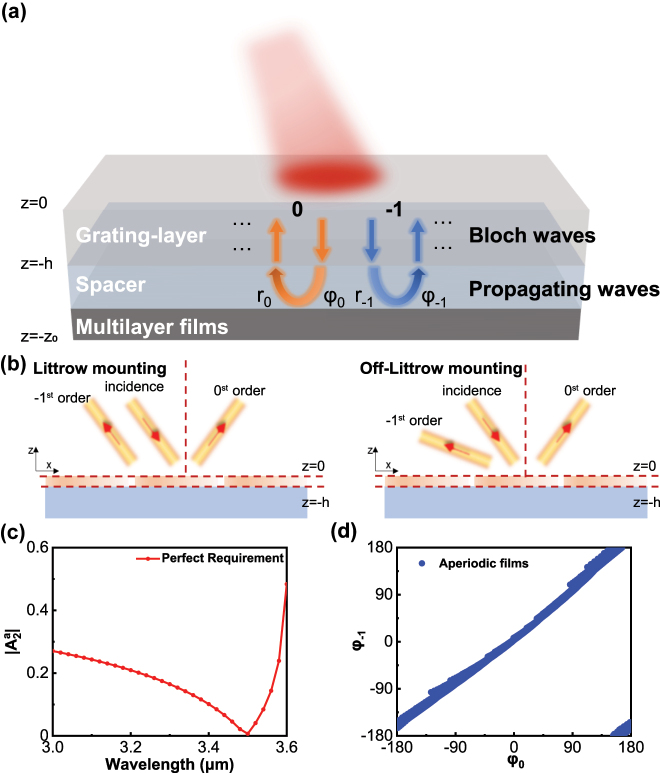
Broadband perfect Littrow diffraction metasurface. (a) The diagram of metasurface system. From top to bottom are grating-layer, spacer and aperiodic multilayer films. (b) Schematic of Littrow mounting and off-Littrow mounting. (c) The relationship between $\left|A_{2}^{a}\right|$ and incident wavelength *λ*. (d) Modulation range of *A* ^*P*^ with periodic mirror and aperiodic films.

Traditional reflective metasurfaces are primarily composed of a metal substrate and metal/dielectric structural layers. For instance, in phase gradient metasurfaces, the primary function of the structural layer is to achieve a 2*π*-phase shift, while the substrate layer provides reflective capabilities. Such metasurfaces lack the ability for longitudinal modulation. In contrast, as a quasi-three-dimensional structure, the broadband perfect Littrow diffraction metasurface consists of an upper grating-layer, an intermediate spacer and lower aperiodic multilayer films, as shown in Figure 1(a). The incident light undergoes diffraction through gratings before entering the spacer, engaging in multiple scattering interactions facilitated by the combined effect of gratings and multilayer films. According to the Huygens-Fresnel principle [32], [33], every point on a wavefront is itself the source of spherical wavelets during the propagation of light, and the secondary wavelets emanating from different points mutually interfere. Therefore, the reflection of the metasurface can be indirectly influenced by controlling the field distribution (energy flow distribution and Poynting vector) on the plane above the metasurface (*z* = 0). Perfect anomalous reflection requires sufficient lateral energy flow transfer capability due to the conservation of energy. Thus, desired lateral energy flow transfer at a specific position (*z* = 0) is a necessary condition.

To facilitate the control of energy flow in different regions, the energy flow of incident light is defined as Bloch waves within the gratings while as propagation waves and evanescent waves within spacer. The lateral energy flow transfer capability of the system is primarily achieved through multiple scattering within the spacer. Evanescent waves are neglected when analyzing as they cannot undergo multiple scattering. Therefore, the energy flow distribution across the whole system can be expressed as:

(1) $$S^{a}\approx S^{B}+S^{P},$$

where *S* ^*a*^, *S* ^*B*^, *S* ^*P*^ are the lateral energy flow of entire metasurface system, Bloch waves and propagation waves. Since there is no energy loss within the metasurface, the energy flow *S*_*z*_ at the bottom of the metasurface (*z* = −*z*_0_) can be defined as 0. We can obtain the relationship of distribution around the metasurface:

(2) $$\begin{array}{ccc} & \;\overset{x_{0}+\Delta x}{\underset{x_{0}}{\int }}S_{z}\left(x,z=0\right)dx= & \overset{0}{\underset{-z_{0}}{\int }}\left[S_{x}\left(x_{0},z\right)-S_{x}\left(x_{0}+\Delta x,z\right)\right]dz, \end{array}$$

where *S*_*z*_ and *S*_*x*_ are the *z* and *x* components of the Poynting vector. In the following sections, all desired energy flow is achieved by the regulation of *S*_*z*_ as an indirect and feasible method.

When the metasurface operates around Littrow mounting, the reflection angle is equal to the incident angle as Figure 1(b). *S* ^*a*^ (*z* = 0) can be obtained by the electromagnetic field distribution at the plane above the metasurface by Sa(x)=12ReEtotal,xHtotal,y* , where *E* represents the electric field and *H* represents the magnetic field. The result is simplified as:

(3) $$S_{1}^{a}=-\frac{1}{2}y_{0}{E_{i}}^{2}r^{0}r^{-1}\cos\theta \cos2Gx,$$

where *G* is the reciprocal lattice vector, *θ* is the incident angle and *r* ^0^, *r* ^−1^ are the amplitudes of reflective channels. When achieving perfect anomalous reflection at the −1st order, which means *r* ^0^ = 0 and *r* ^−1^ = 1, the condition for perfect anomalous reflection is simplified as:

(4) $$S_{1}^{a}=S_{1}^{B}+S_{1}^{P}=0,$$

(5) $$\left|A_{1}^{a}\right|=0,$$

where $A_{1}^{a}$ is the amplitude of the total energy flow. Similarly, the energy flow of propagation waves $S_{1}^{P}$ can be obtained by the electromagnetic field distribution at *z* = *−h*. Due to the reflectance of the Bragg reflector is 100 % without transmission and absorption losses, $S_{1}^{P}$ always equal to zero around Littrow mounting, which means there is no transferred energy. In other words, the requirement of perfect anomalous reflection around Littrow mounting can be written as:

(6) $$S_{1}^{a}=S_{1}^{B}=S_{1}^{P}=0.$$

When the incident wavelength changes, the reflection angle will deviate from the Littrow angle as Figure 1(b). Traditional all-dielectric metasurfaces attribute the efficiency decrease to insufficient dispersion capability. It is not straightforward to analyze based solely like Littrow mounting, as the energy flow distribution changes with the alteration of the electromagnetic field. The total energy flow $S_{2}^{a}$ a in this situation is as:

(7) $$S_{2}^{a}=\frac{1}{2}y_{0}{E_{i}}^{2}\left[\begin{array}{c} r^{-1}\left(\cos\theta_{0}-\cos\theta_{1}\right)\cos\left(2Gx+\tau_{1}\right)\\ -r^{0}r^{-1}\left(\cos\theta_{0}+\cos\theta_{1}\right)\cos\left(2Gx+\tau_{2}\right) \end{array}\right],$$

where *θ*_0_ is the reflection angle of 0th order, *θ*_1_ is the reflection angle of −1st order. When achieving perfect anomalous reflection at the −1st order, which means *r* ^0^ = 0 and $r^{-1}=\sqrt{\frac{\cos\theta_{0}}{\cos\theta_{1}}}$ , the condition for perfect anomalous reflection is simplified as:

(8) $$S_{2}^{a}=\frac{1}{2}y_{0}{E_{i}}^{2}\sqrt{\frac{\cos\theta_{0}}{\cos\theta_{1}}}\left(\cos\theta_{0}-\cos\theta_{1}\right)\cos\left(2Gx+\tau_{1}\right),$$

(9) $$\left|A_{2}^{a}\right|=\frac{1}{2}y_{0}{E_{i}}^{2}\sqrt{\frac{\cos\theta_{0}}{\cos\theta_{1}}}\left(\cos\theta_{0}-\cos\theta_{1}\right).$$

The amplitude $A_{2}^{a}$ is decided by reflection angle. According to the grating formula, the relationship between $\left|A_{2}^{a}\right|$ and incident wavelength is shown in Figure 1(c). $S_{2}^{P}$ can be obtained by the electromagnetic field distribution at *z* = −*h*, which is simplified as:

(10) $$S_{2}^{P}=A_{2}^{P}\cos\left(2Gx+\tau^{P}\right),$$

(11) $$A_{2}^{P}=\frac{1}{2}y_{0}{E_{i}}^{2}c_{0}^{-}c_{-1}^{-}\left[\begin{array}{c} \left(\cos\theta_{0}+\cos\theta_{1}\right)\sin\frac{\Phi_{0}-\Phi_{1}}{2}\\ +\left(\cos\theta_{0}-\cos\theta_{1}\right)\sin\frac{\Phi_{0}+\Phi_{1}}{2} \end{array}\right],$$

where *θ*_0_ and *θ*_1_ are deflection angles in the spacer, Φ_0_ and Φ_1_ are the reflection phase of 0th and −1st order propagating waves provided by the spacer and aperiodic films (Supplementary Material S_I_). Evidently, Φ_0_ and Φ_1_ play a crucial role in influencing the resonance interference conditions of distinct optical paths, as shown in Figure 1(b), so that $A_{2}^{P}$ can be controlled by adjusting the thickness of the spacer and aperiodic films. As shown in Figure 1(d), the modulation range of the propagating wave is limited, mainly because when the incident angle approaches the diffraction angle, Φ_0_ and Φ_1_ are similar. According to Supplementary Material S_I_, $A_{2}^{P}$ can never meet the requirements of $A_{2}^{a}$ . Therefore, it is difficult to independently modulate the propagation wave to meet the requirements of the total energy flow. Further modulation of the Bloch waves is necessary to enhance lateral energy flow transfer capability.

Bloch waves propagate within the upper structure, and the field distribution can be obtained through the rigorous coupled-wave analysis (RCWA) [34], [35], which can be written as [36]:

(12) $$E_{x}=\underset{m}{\sum }S_{m}\left(z\right){\text{e}}^{\mathrm{jGmx}},$$

(13) $$H_{y}=\sqrt{\frac{\varepsilon_{0}}{\mu_{0}}}\underset{m}{\sum }U_{m}\left(z\right){\text{e}}^{\mathrm{jGmx}},$$

where *m* is the order of Bloch waves. *S*_*m*_(*z*) and *U*_*m*_(*z*) include the downward and upward waves. The field distribution of Bloch waves is primarily influenced by structural parameters [37]. Phase discontinuities can be controlled by varying the height, width, and spacing of the structure. Boundary conditions indirectly affect the field distribution, and adjusting the spacer as well as the topmost bilayer aperiodic films can alter the reflection phase. Since the underlying film layers maintain the form of a Bragg reflector, a 100 % reflectance can still be preserved.

By simultaneously regulating both the propagating wave and the Bloch wave, the total energy flow can satisfy Equation (8). At this point, the energy of the reflected light is concentrated at a specific angle. Perfect anomalous reflection at any angle can be achieved through energy flow manipulation, making it a versatile method that is not constrained by the incidence conditions (e.g., Littrow-mounting). As previously mentioned, to enhance broadband efficiency, it is necessary to meet the requirements of *S* ^*a*^ under both Littrow mounting and off-Littrow mounting. In other words, achieving broadband perfect anomalous reflection is possible when both Equations (4) and (8) are simultaneously satisfied.

During the design process, we first kept the thickness of the multilayer films constant and scanned the structure’s linewidth and height, with the goal of finding the set of parameters that best matches the target energy flow. After selecting this set of parameters as the initial solution, we applied particle swarm optimization (PSO) to the aperiodic film thickness and structural linewidth to obtain the final design result. This approach not only aligns with the theory of controlling non-local responses through energy flow modulation, as discussed earlier, but also helps prevent the final result from falling into a local optimum.

In summary, within the metasurface, the multilayer film plays a primary role in the propagation wave energy flow *S* ^*P*^, while the upper structure governs the Bloch wave energy flow *S* ^*B*^. Under strict Littrow-mounting, where the incident angle equals the diffraction angle, the propagation wave energy flow remains zero (*S* ^*P*^ = 0), and only the Bloch wave affects the total energy flow. Therefore, in the design process, we initially do not consider changes in the thickness of the multilayer films. When deviating from Littrow-mounting, the propagation wave energy flow *S* ^*P*^ gradually increases, but its control ability is limited, with the Bloch wave *S* ^*B*^ still playing a decisive role. Therefore, to achieve perfect diffraction in the −1st order, under Littrow-mounting, the Bloch wave *S* ^*B*^ needs to avoid the generation of non-local effects, maintaining its lateral energy flow at zero (*S* ^*B*^ = 0). When deviating from the Littrow-mounting, the metasurface is influenced by both the propagation wave *S* ^*P*^ and Bloch wave *S* ^*B*^, requiring the enhancement of non-local effects to meet the perfect diffraction requirements.

## 3 Results

### 3.1 Broadband reflectors based on periodic gratings

**Figure 2:**
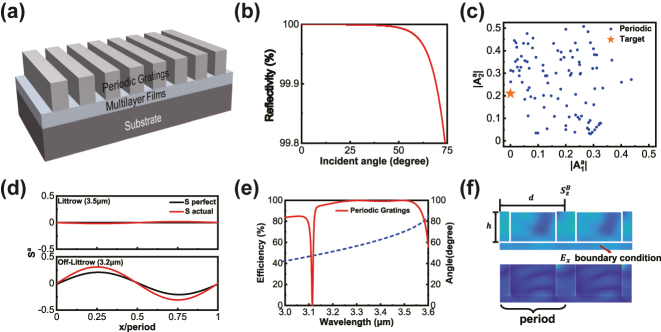
Broadband reflectors based on periodic gratings. (a) Schematic of broadband reflectors based on periodic gratings. (b) The reflectance of multilayer films ensures 100 % at different incident angles within a broadband range. (c) Scanning results for different parameters of periodic gratings. (d) Energy flow *S* ^*a*^ for 3.5 µm and 3.2 µm incidents. (e) The calculated efficiency and diffraction angle of the broadband reflector based on periodic gratings. (f) Schematic of Poynting vector and electric field in the periodic gratings.

We initially designed a reflector with an upper structure consisting of periodic gratings as shown in Figure 2(a). 3.5 µm mid-infrared laser was incident at 70°, necessitating a sufficiently small grating period to ensure only 0th and −1st diffraction orders. The period was set to 1.86 µm. On the substrate, 8 sets of Si/SiO_2_ multilayer films were stacked, with the thickness of the top two layers being freely adjustable within a certain range. The aperiodic multilayer films ensure 100 % reflectance at different angles (as shown in Figure 2(b)) while providing a certain degree of control over Φ_0_ and Φ_1_. The spacer prevents multiple scattering of evanescent waves. The thickness range of the spacer and aperiodic multilayer films was preliminarily determined to simplify the design of the upper structure. Structural parameters of the periodic grating, including height *h* and width *d*, aim to obtain a set of parameters allowing the entire metasurface system to meet the requirements of the total energy flow *S* ^*a*^ over a broadband range (3.2 µm ∼ 3.5 µm).

By scanning the parameters *h* and *d*, we computed *A* ^*a*^ for 3.5 µm and 3.2 µm incidents, as shown in the Figure 2(c). The parameters yielding values closest to the target were selected as the initial solution. We utilized PSO (particle swarm optimization [38]) to optimize the thickness of the spacer layers and aperiodic multilayer films within a specified range, ultimately achieving a complete quasi-three-dimensional reflector based on periodic gratings. After PSO, designed periodic gratings structure on aperiodic multilayer films were obtained and the energy flow was simulated by RCWA. In this set of structural parameters, $S_{1}^{a}$ for 3.5 µm incidence closely matches the requirements for perfect anomalous reflection. However, for 3.2 µm incidence, there is a deviation between $S_{2}^{a}$ and the desired energy flow as shown in Figure 2(d). In theory, the deviation results in a rapid decrease in efficiency around 3.2 µm incident. Figure 2(e) shows the calculated efficiency of reflector based on periodic gratings in the range of 3.24 µm–3.52 µm, with 99 % efficiency.

The structural constraints of periodic gratings make it challenging to fulfill the requirement of achieving a broadband reflector with an efficiency consistently exceeding 99 %. We attribute this to the insufficient non-local response of the periodic grating, which results in reduced efficiency when the incident wavelength deviates from Littrow mounting. The magnitude of the Bloch energy flow can indirectly reflect the strength of the non-local response, and it is influenced by both the structural parameters and the boundary conditions, as shown in Figure 2(f). Periodic gratings are more sensitive to boundary conditions under large-angle incidence, which are determined by the aperiodic multilayer films and have a very limited range of adjustment. Therefore, a new structure is required, where the structural parameters play a decisive role in modulating Bloch wave propagation.

### 3.2 Broadband reflectors based on supercell metagratings

**Figure 3:**
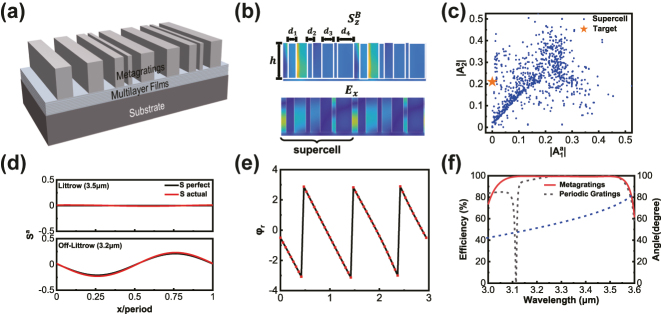
Broadband reflectors based on supercell metagratings. (a) Schematic of broadband reflectors based on supercell structures. (b) Schematic of Poynting vector and electric field in the supercell structures. (c) Scanning results for different parameters of supercell structures. (d) Energy flow *S* ^*a*^ for 3.5 µm and 3.2 µm incidents. (e) The reflection phase of the entire metasurface system. (f) The calculated efficiency and diffraction angle of the broadband reflector based on supercell structures. The dashed line represents the efficiency curve of the aforementioned periodic grating.

The fundamental design principle of a metasurface is based on the assumption of locality, where each subunit shares the same environment. In practice, within a non-local environment, the scattering at a given position depends on the fields or structures in the distant region, especially when neighboring subunit structures undergo drastic variations. Therefore, the metasurface with a supercell structure on the upper layer is designed to enhance the control over Bloch waves, as shown in Figure 3(a). The structures consist of four subunits, each having a grating structure with the same height but varying widths. The coupling between these subunits induces non-local effects [39], influencing Bloch waves. By designing the parameters of the grating structures, we can simulate this effect and indirectly modulate the energy flow. The supercell structures increases the structural degrees of freedom [40], as shown in Figure 3(b), thereby mitigating the impact of lower boundary conditions on Bloch wave propagation.

By scanning the subunit grating widths *d*_1_, *d*_2_, *d*_3_, *d*_4_ and height *h* (with ranges determined by fabrication capabilities), we calculated the amplitudes *A* ^*a*^ for 3.5 µm and 3.2 µm incidences, as shown in Figure 3(c). The results exhibited a significantly expanded range, perfectly covering the target values. After selecting the structures parameters closest to the target value (results can be found in the Supplementary Materials), PSO can be employed to determine the thickness of the aperiodic multilayer films, achieving the highest broadband efficiency. In this set of structural parameters, the actual values of *S* ^*a*^ for both 3.5 µm and 3.2 µm incidences closely match the requirements for perfect anomalous reflection, as shown in Figure 3(d). To validate the correctness of the results, we employed finite-difference time-domain (FDTD) simulations to calculate the reflection phase of the entire metasurface system, as depicted in Figure 3(e). At the designed wavelength, the metasurface system consistently maintains a phase gradient of 2*π*, demonstrating broad and high-efficiency performance. Meanwhile, a broadband perfect anomalous reflector in the range of 3.11 µm–3.52 µm was simulated by RCWA, as shown in Figure 3(f), with 99 % efficiency. Compared to the reflector based on periodic gratings, the bandwidth (efficiency exceeding 99 %) increased by 46 %. The results are consistent with the design theory discussed earlier, where broadband perfect diffraction can be achieved by controlling the energy flow at two specific wavelengths. As the bandwidth increases and the incident wavelength deviates from the Littrow wavelength, the lateral energy flow required for perfect diffraction gradually increases. Through verification at multiple points within the design bandwidth (Supplementary Material S2), we observed that the energy flow of the designed metasurface also gradually increases as the incident wavelength deviates. When we can satisfy the perfect diffraction conditions at two specific wavelengths (3.5 µm and 3.2 µm), the energy flow within the bandwidth between them varies within a certain range (0 $∼S_{2}^{a}$ ).

**Figure 4:**
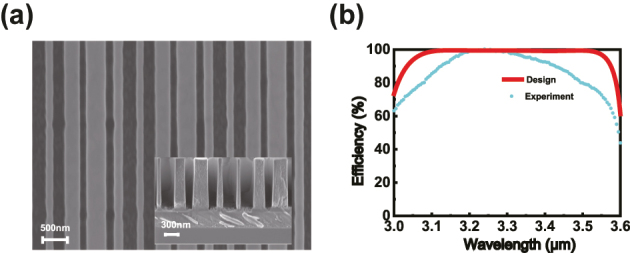
Fabrication and characterization of the broadband metasurface. (a) SEM side view image and cross-sectional image of the sample. (b) The experimental −1st order relative efficiency and the theoretical design efficiency.

Samples were prepared using a multilayer film deposition process and microstructure preparation techniques. The detailed sample preparation process is provided in the Materials and Methods section. Cross-sectional images of the samples were obtained through scanning electron microscopy (SEM), as shown in Figure 4(a), and their parameters matched the design specifications. Spectral tests were conducted to observe effective suppression of reflection from other orders within the designed wavelength range of 3.11 µm–3.52 µm. The results of the detailed spectral tests are discussed in the Sample Characterization section. The broadband anomalous reflection efficiency obtained from the experiment is illustrated in Figure 4(b). The good consistency observed indicates the reliability of our manufacturing and testing results.

## 4 Discussion

**Figure 5:**
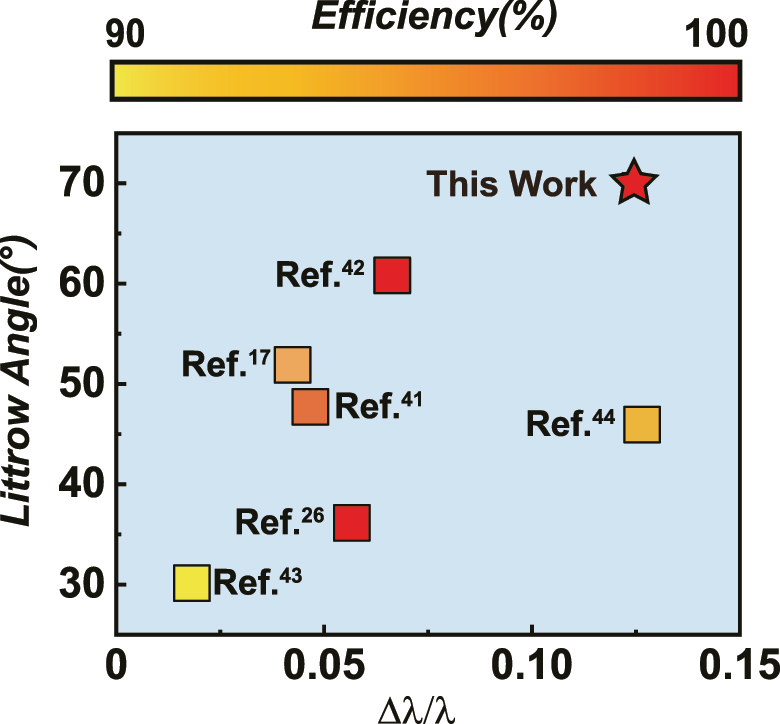
Comparison of research on Littrow diffraction devices [17], [26], [41], [42], [43], [44]. The *x*-axis represents the ratio of bandwidth to central wavelength, the *y*-axis represents the Littrow angle, and the color bar represents the diffraction efficiency.

A non-local metasurface composed of supercell structures and aperiodic multilayer films has been successfully designed, achieving broadband perfect Littrow diffraction under 70° incidence. We enhance or suppress non-local responses at different wavelengths in order to meet broadband requirements under large-angle incidence. A comparison of the results with recent research on Littrow diffraction reveals that our design non-local metasurface offers advantages in terms of incidence, bandwidth, and efficiency, as shown in Figure 5. In fact, as the design wavelength increases, selecting appropriate metasurface materials and fabrication processes becomes increasingly challenging. Currently, no studies have demonstrated broadband perfect Littrow diffraction under large-angle incidence in the mid-infrared band.

The broadband Littrow diffraction system in the article operates in the vicinity of Littrow-mounting. However, Littrow mode is just one specific functionality of the reflective element. Through the modulation of non-local responses, it is possible to achieve arbitrary angles of deflection of light. In other words, incident light at any angle can be perfectly anomalously reflected to any desired angle.

In summary, broadband perfect Littrow diffraction devices under large-angle incidence are currently in high demand as optical elements and will continue to be so in the future. The analysis and modulation of non-local responses represent effective means for designing such metasurfaces. The supercell structures effectively enhance the lateral transfer capability of energy flow, and more complex upper metasurface structures allow for more precise control. For instance, two-dimensional structures can achieve depolarization. Our work provides significant advancements for the application of metasurfaces in fields such as laser radar, spectral analysis, imaging systems, and beyond.

## 5 Materials and methods

### 5.1 Numerical simulation

An open-source rigorous coupled-wave analysis (RCWA) solver, “RETICOLO” [45], was used to perform the full-wave simulation including the calculation of diffraction efficiency, Poynting vector, reflection phase, and electromagnetic field distribution. One-dimensional RCWA simulation with a period of 1.86 µm in the *x*-direction, refractive indices of 3.40 for Si, and 1.47 for SiO_2_. The system employs a plane wave with TM polarization incident at 70°. The structural parameters are obtained through particle swarm optimization, typically converging within 400 iterations.

### 5.2 Sample fabrication

The Si/SiO_2_ multilayer films were initially deposited on a Si substrate through ion beam sputtering (IBS). Following the deposition of 7 sets of periodic reflection layers, 2 aperiodic layers, and a spacer, the top structural layer is an 1,100 nm silicon layer, fabricated using electron-beam deposition to facilitate etching. Subsequently, a layer of positive electron-beam resist (AR-P 6200.13) was spin-coated onto the upper Si layer. Electron beam lithography (EBL) was performed using the Raith EBPG 5200 system to define the inverse pattern of the target structure on the resist. After exposure, the resist was developed in ethyl acetate. Furthermore, we employed inductively coupled reactive ion etching with an etching rate of approximately 2.75 nm/s, using CHF_3_ and SF_6_ gases. Following the etching process, any remaining photoresist was removed using an oxygen plasma. The detailed experimental parameters can be found in the Supplementary Material.

### 5.3 Sample characterization

Directly measuring the −1st order diffraction efficiency is challenging, as there is virtually no angle between the incident light and the −1st order diffracted light under Littrow-mounting. We indirectly determine the −1st order diffraction efficiency by measuring the energy of the 0th order reflection using PHOTON RT Spectrophotometer. This method allows for relative efficiency measurements, as the designed metasurface produces only the 0th and −1st orders during diffraction. The detailed experiment process can be found in the Supplementary Material.

## Supplementary Material

Supplementary Material Details
