# Supplementary material for: Broadband perfect Littrow diffraction metasurface under large-angle incidence
Source: Nanophotonics. 2025 Feb 10;14(8):1221–9. doi: 10.1515/nanoph-2024-0622 (PMC12019931; doi:10.1515/nanoph-2024-0622)
Supplement: Supplementary file 1 — Supplementary Material Details [file j_nanoph-2024-0622_suppl_001.pdf]

Jingyuan Zhu†,Siliang Zhou†,Tao He,Chao Feng,Zhanshan Wang,Siyu Dong*,Xinbin Cheng*

Supplementary material: Broadband Perfect Littrow Diffraction Metasurface under Large-Angle Incidence

Contents

S_I. Detailed derivation of the ideal energy-flow distribution

S_{II}. Detailed design of supercell structures

S_{III}. Tolerance in the design of the structures

S_{IV}. Detailed process of sample fabrication

S_V. Detailed process of experimental characterization

Jingyuan Zhu,Siliang Zhou,Tao He,Chao Feng,Zhanshan Wang,Siyu Dong*,Xinbin Cheng*, Institute of Precision Optical Engineering, School of Physics Science and Engineering, Tongji University, Shanghai 200092, China; MOE Key Laboratory of Advanced Micro-Structured Materials, Shanghai 200092, China; Shanghai Frontiers Science Center of Digital Optics, Shanghai 200092, China; Shanghai Professional Technical Service Platform for Full-Spectrum and High-Performance Optical Thin Film Devices and Applications, Shanghai 200092, China; Shanghai Institute of Intelligent Science and Technology, Tongji University, Shanghai 200092, China.

S_I. Detailed derivation of the ideal energy-flow distribution

In the general case, when TM waves are incident at an angle of 70° onto the metasurface, due to the subwavelength periodicity, the incident wave will result in scattering in two channels: 0th order and -1st order.

When Littrow mounting, the diffraction angle is equal to the incident angle θ. Then, the electromagnetic field at $z = 0$ plane can be written as:

$$\begin{aligned} E_{total,x=}&E_i(e^{+jGx} - r^0 e^{+jGx+j\phi^0} - r^{-1}e^{-jGx+j\phi^{-1}})\cos\theta \\ H_{total,y} &= y_0 E_i(e^{+jGx} + r^0 e^{+jGx+j\phi^0} + r^{-1}e^{-jGx+j\phi^{-1}}) \end{aligned} \tag{S1}$$

where r^0 and r^{-1} are reflection coefficients of whole metasurfaces system, and $(r^{-1})^2 + (r^0)^2 = 1$ satisfies energy conservation. G is the reciprocal lattice vector, and equals to $k_0 \sin\theta$. According to the electromagnetic field, we can obtain the actual energy flow distribution through $S^a(x) = \frac{1}{2}\,\mathrm{Re}(E_{total,x}H^*_{total,y})$, which can be written as:

$$\begin{aligned} S_1^a &= \frac{1}{2}\,\mathrm{Re}\left(E_{\mathrm{total},x}H^*_{\mathrm{total},y}\right) \\ &= \frac{1}{2} y_0 E_i^2\,\mathrm{Re}\begin{bmatrix} \cos\theta + r^0 e^{-j\varphi^0}\cos\theta + r^{-1}e^{+2jGx-j\varphi^{-1}\cos\theta} \\ -r^0\cos\theta e^{j\varphi^0} - (r^0)^2\cos\theta \\ -r^{-1}\cos\theta e^{-jGx+j\varphi^{-1}} - r^0 r^{-1}\cos\theta e^{2jGx+j(\varphi^0-\varphi^{-1})} \\ -r^{-1}\cos\theta e^{-2jGx+j\varphi^{-1}} - r^0 r^{-1}\cos\theta e^{-2jGx-j(\varphi^0-\varphi^{-1})} - (r^{-1})^2\cos\theta \end{bmatrix} \\ &= -\frac{1}{2} y_0 E_i^2 r^0 r^{-1}\cos\theta\cos 2Gx \end{aligned} \tag{S2}$$

When achieving perfect anomalous reflection, $r^0 = 0$, $r^{-1} = 1$. Therefore, the energy flow distribution for perfect anomalous reflection can be expressed as $r^{-1} = 1$.

Meanwhile, the electromagnetic field at $z = -h$ plane can be written as:

$$\begin{aligned} E_x &= E_i\left[c_0^-\left(1 - r_0^- e^{j\Phi_0}\right)e^{+jGx}\cos\theta + c_{-1}^-\left(1 - r_1^- e^{j\Phi_1}\right)e^{-jGx}\cos\theta\right] \\ H_y &= y_0 E_i\left[c_0^-\left(1 + r_0^- e^{j\Phi_0}\right)e^{+jGx} + c_{-1}^-\left(1 + r_1^- e^{j\Phi_1}\right)e^{-jGx}\right] \end{aligned} \tag{S3}$$

where c_0^- and c_{-1}^- are transmission coefficient of the metagratings, r_0^- and r_{-1}^- are reflection coefficients of the Bragg reflector. Due to the reflectance of the Bragg reflector is 100% without transmission and absorption losses, $r_0^- = r_1^- = 1$, $\Phi_0 = \Phi_1$. According to the electromagnetic field, we can obtain the actual energy flow of propagation waves, which can be written as:

$$S_1^P = \frac{1}{2}\,\mathrm{Re}\left(E_x H_y^*\right) = \frac{1}{2} y_0 {E_i}^2 \cos\theta\,\mathrm{Re}\begin{bmatrix} (c_0^-)^2\left(-e^{j\Phi_0} + e^{-j\Phi_0}\right) \\ +c_0^- c_{-1}^-\left(e^{-j\Phi_0} - e^{j\Phi_0} + e^{-j\Phi_1} - e^{j\Phi_1}\right) \\ +(c_{-1}^-)^2\left(-e^{j\Phi_1} + e^{-j\Phi_1}\right) \end{bmatrix} = 0 \tag{S4}$$

which means the energy flow of propagation waves always equals zero when Littrow mounting.

When Off-Littrow mounting, the diffraction angle θ_1 is different from the incident angle θ_0. Then, the electromagnetic field at z=0 plane can be written as:

$$\begin{aligned} E_{total,x}&=E_i(\cos\theta_0 e^{+jGx} - r^0\cos\theta_0 e^{+jGx+j\phi^0} - r^{-1}\cos\theta_1 e^{-jGx+j\phi^{-1}}) \\ H_{total,y} &= y_0 E_i(e^{+jGx} + r^0 e^{+jGx+j\phi^0} + r^{-1}e^{-jGx+j\phi^{-1}}) \end{aligned} \quad \text{(S5)}$$

$(r^{-1})^2\cos\theta_1 + (r^0)^2\cos\theta_0 = \cos\theta_0$ satisfies energy conservation. G is the reciprocal lattice vector, and equals to $k_0\sin\theta$. When achieving perfect anomalous reflection, $r^0 = 0$, $r^{-1} = \sqrt{\frac{\cos\theta_0}{\cos\theta_1}}$. Therefore, the energy flow distribution for perfect anomalous reflection can be written as:

$$S_2^a = \frac{1}{2}\mathrm{Re}\left(E_{\mathrm{total},x}H^*_{\mathrm{total},y}\right) = \frac{1}{2}y_0E_i^2\sqrt{\frac{\cos\theta_0}{\cos\theta_1}}\mathrm{Re}\begin{bmatrix}\cos\theta_0 e^{+2jGx-j\varphi^{-1}} \\ -\cos\theta_1 e^{-2jGx+j\varphi^{-1}}\end{bmatrix} \quad \text{(S6)}$$

$$A_2^a = \frac{1}{2}y_0E_i^2\sqrt{\frac{\cos\theta_0}{\cos\theta_1}}(\cos\theta_0 - \cos\theta_1) \quad \text{(S7)}$$

where A_2^a is the amplitude of energy flow.

Meanwhile, the electromagnetic field at z=-h plane can be written as:

$$\begin{aligned} E_x &= E_i\left[c_0^-\left(1-r_0^-e^{j\Phi_0}\right)e^{+jGx}\cos\theta_0 + c_{-1}^-\left(1-r_1^-e^{j\Phi_1}\right)e^{-jGx}\cos\theta_1\right] \\ H_y &= y_0E_i\left[c_0^-\left(1+r_0^-e^{j\Phi_0}\right)e^{+jGx} + c_{-1}^-\left(1+r_1^-e^{j\Phi_1}\right)e^{-jGx}\right] \end{aligned} \quad \text{(S8)}$$

Due to the reflectance of the Bragg reflector is 100% without transmission and absorption losses, $r_0^- = r_1^- = 1$. Therefore, the actual energy flow of propagation waves can be written as:

$$S_2^P = \frac{1}{2}\mathrm{Re}\left(E_xH_y^*\right) = \frac{1}{2}y_0{E_i}^2c_0^-c_{-1}^-\mathrm{Re}\begin{bmatrix}\left(1-e^{j\Phi_1}\right)\left(1+e^{-j\Phi_0}\right)e^{-2jGx}\cos\theta_1 \\ +\left(1-e^{j\Phi_0}\right)\left(1+e^{-j\Phi_1}\right)e^{+2jGx}\cos\theta_0\end{bmatrix} \quad \text{(S9)}$$

$$A_2^P = \frac{1}{2}y_0{E_i}^2c_0^-c_{-1}^-\begin{bmatrix}(\cos\theta_0+\cos\theta_1)\sin\frac{\Phi_0-\Phi_1}{2} \\ +(\cos\theta_0-\cos\theta_1)\sin\frac{\Phi_0+\Phi_1}{2}\end{bmatrix} \quad \text{(S10)}$$

Through calculations, it is difficult to independently modulate the propagation wave S_2^P to meet the off-Littrow mounting requirements of the total energy flow S_2^a.

Since the incident conditions in this paper are around Littrow incidence, the incident angle is almost equal to the diffraction angle. As shown in Figure 1(d), the phases ϕ_0 and ϕ_1 are also nearly equal at this point (ϕ_0=ϕ_1). From equations (S7) and (S10), it can be derived that:

$$\left|\frac{A_2^P}{A_2^a}\right| = c_0^-c_{-1}^-\sin\phi\sqrt{\frac{\cos\theta_1}{\cos\theta_0}} < 1 \quad \text{(S11)}$$

Since the phase difference can be neglected, A_2^P can never meet the requirements of A_2^a.

S_{II}. Detailed design of supercell stuctures

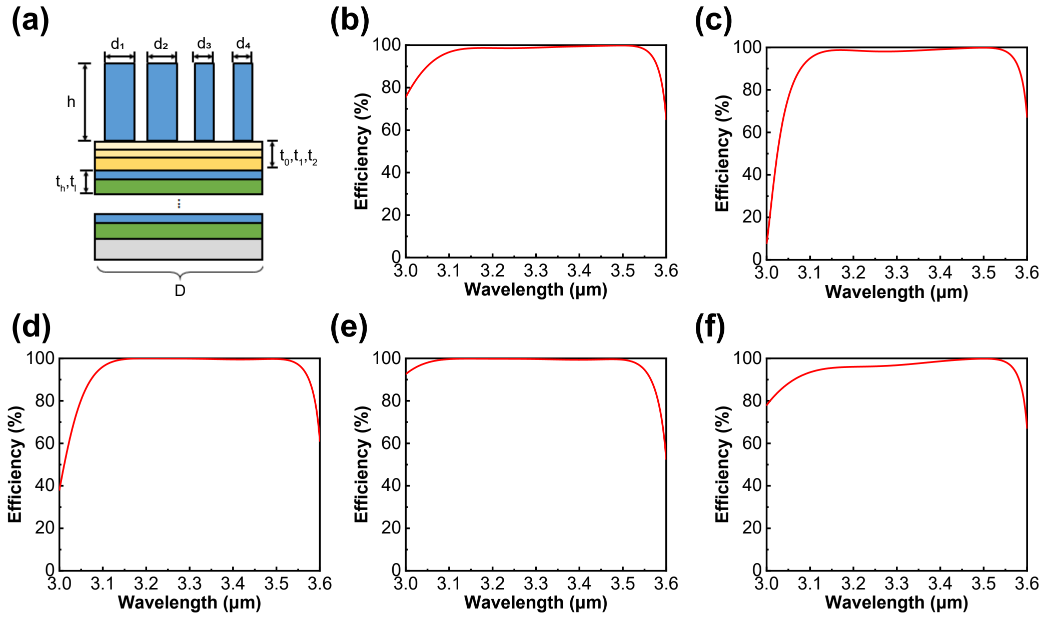

Fig. S1: Diffraction efficiency curves of different initial parameters. (a) Structure Parameter Schematic Diagram (b)∼(f) Height and width of subunit gratings are listed as followed, (b): 1300nm, 419nm, 233nm, 140nm, 47nm, (c): 1100nm, 419nm, 233nm, 140nm, 326nm, (d): 1100nm, 419nm, 326nm, 233nm, 47nm, (e): 1300nm, 419nm, 140nm, 47nm, 140nm, (f): 1500nm, 419nm, 140nm, 47nm, 47nm.

When designing broadband metasurface, we utilize supercell structures to excite non-local effects[1], enhancing the modulation capability of Bloch waves. With a fixed period(1.86µm) and sub-period(465nm), differences in parameters between adjacent structures affect the distribution of Bloch wave energy flow. Theoretically, the greater aspect ratio difference between adjacent structures leads to stronger non-local effects. Among the scanning results, we selected several sets of structural parameters that closely matched the target energy flow as initial solutions. The specific parameters and their corresponding efficiency curves are shown in Figure S1.

Due to limitations in the fabrication capabilities of Si, we selected (b) as the initial solution for optimization. 7 sets of Si/SiO_2 multilayer films with alternating high and low refractive indices are employed to achieve close to 100% reflectance. The thickness of each layer (t_h and t_l) is chosen to be a quarter-wavelength optical thickness, i.e., $\lambda/4n_{high}$ and $\lambda/4n_{low}$, where n_{high} and n_{low} are the refractive indices of the high and low-index materials, respectively. The aperiodic films thickness depends on the phase and the upper-layer structure. The spacer needs to be sufficiently thick to prevent the scattering of evanescent waves at a 70° oblique incidence. The final obtained structural parameters are listed in Table S1.

By analyzing the energy flow at multiple points within the design bandwidth, we found that as the bandwidth increases and the incident wavelength deviates from the Littrow wavelength, the lateral energy flow required for perfect diffraction gradually

Tab. 1: Parameters of supercell structures.

h	d_1	d_2	d_3	d_4
	Si	Si	Si	Si
1100nm	207nm	116nm	302nm	419nm
t_0	t_1	t_2	t_h	t_l
SiO_2	Si	SiO_2	Si	SiO_2
119nm	335nm	634nm	258nm	596nm

increases, as shown in Figure S2(a). At the same time, the lateral energy flow generated by the metasurface also varies within a certain range, as shown in Figure S2(b). The results demonstrate that the designed metasurface exhibits broadband high-efficiency performance.

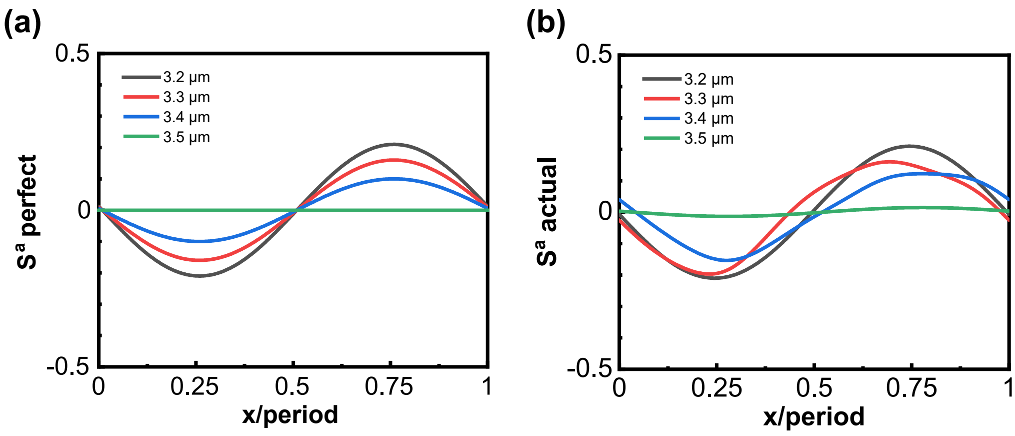

Fig. S2: (a) The trend of the change in the energy flow requirement for perfect diffraction as the incident wavelength varies. (b) The trend of the change in the actual energy flow obtained by the metasurface as the incident wavelength varies.

S_{III}. Tolerance in the design of the structures

To validate the theoretical accuracy and the feasibility of fabrication, we employed the Monte Carlo method to calculate the tolerance of design parameters[2]. After 500 iterations, the results are shown in Figure S3. (a) demonstrates that film thicknesses within a ±20nm error range can maintain efficiency above 98%. (b) shows that material refractive indices within a ±0.1 range can maintain efficiency above 97%. Since the subunit structures have different widths, we calculated the tolerance in percentage terms, as shown in (c), indicating that structure widths within ±10% can maintain efficiency above 97%.

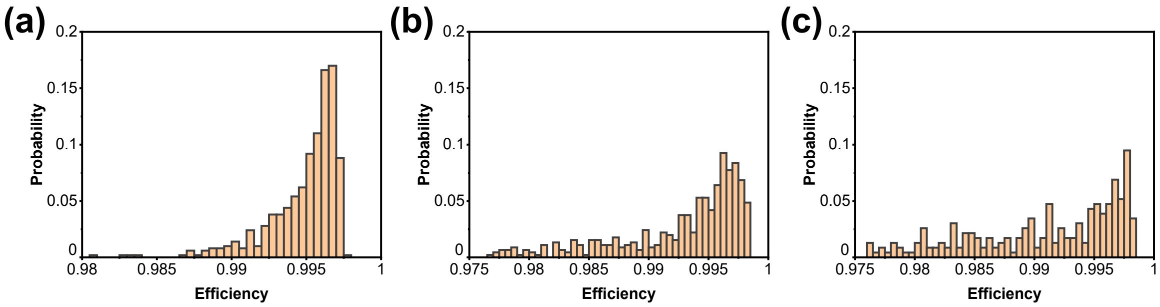

Fig. S3: Tolerance in the design of the structure. (a) Efficiency probability with tolerance (<20nm) for different film thicknesses. (b) Efficiency probability with tolerance (<0.1) for material refractive indices. (c) Efficiency probability with tolerance (<10%) for structure widths.

S_{IV}. Detailed process of sample fabrication

A silicon wafer was used as the substrate, with the metasurface fabricated on it. The sample fabrication process primarily involves physical vapor deposition(PVD), electron beam lithography(EBL), and plasma etching(ICP-RIE), as shown in Figure S4.

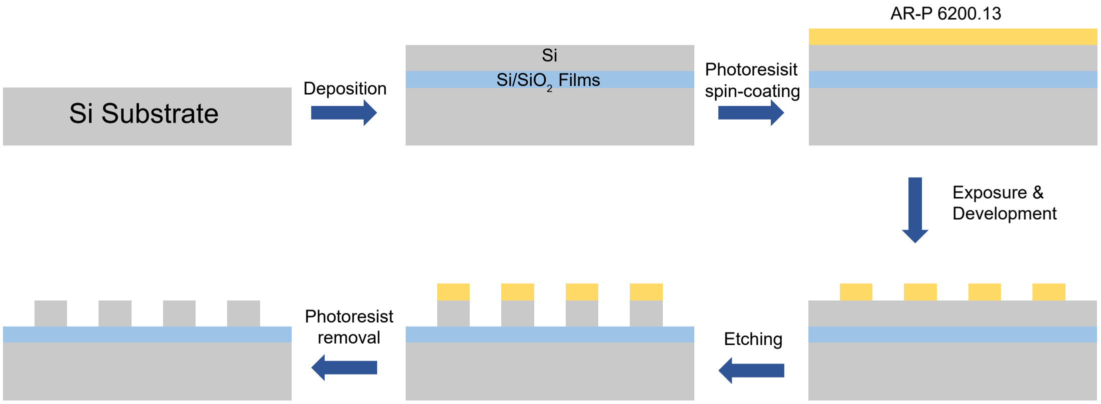

Fig. S4: Schematic of the major fabrication steps of the metasurface.

Step 1: 7 sets of periodic reflection layers, two aperiodic film layers, and one spacer layer were deposited on a clean silicon wafer using IBS (Ion Beam Sputtering). The films deposited by IBS exhibit a denser structure, which effectively prevents the delamination of the multilayer films. The top grating structure layer was fabricated using electron beam deposition. Compared to IBS deposition, the films obtained through electron beam deposition are more suitable for etching high aspect ratio structures.

Step 2: A layer of Surpass4000 was spin-coated as an adhesive for surface modification. Then, electron-beam resist (AR-P 6200.13) with a thickness of approximately 485nm is spin-coated at a rotation speed of 2500 rpm, serving as a mask. The sample is then baked at 180°C for 5 minutes.

Step 3: The pattern was written using a 100keV electron beam lithography (EBPG5200, Raith) system. The beam current was 3nA and the minimum exposure dose was 260μC/cm^2, where narrower slits may require a higher exposure dose. After exposure, the resist is developed in ethyl acetate for 1 minute and fixed in isopropyl alcohol for 30 seconds.

Step 4: we employ inductively coupled reactive ion etching by Oxford PlasmaPro 100 Cobra 300. The spacer layer functions as an insulating layer to ensure that etching occurs exclusively on the Si layer. The flow rates of the SF_6 and CHF_3 gases, working pressure, ICP power, HF power, and temperature were maintained at 15sccm and 45sccm, 20mTorr, 1200W, 25W, and 5°C, respectively. The etching rate was approximately 2.5nm/s.

Step 5: Following the etching process, any remaining photoresist is removed using an oxygen plasma.

S_V. Detailed process of experimental characterization

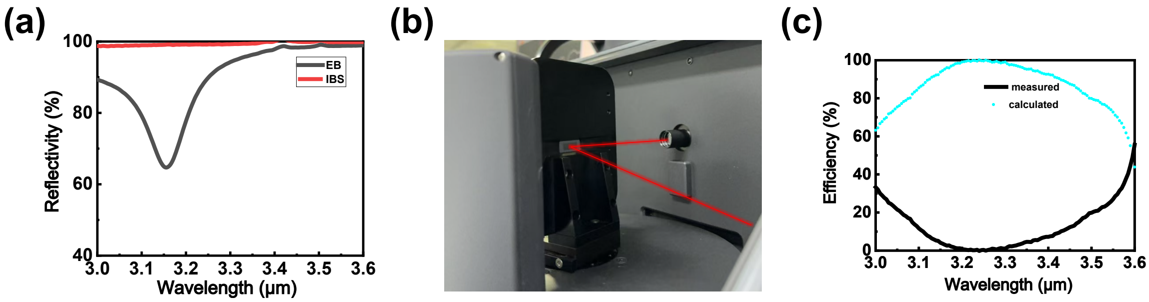

Fig. S5: (a) The reflectivity curve of the multilayer films. (b) Schematic diagram of the spectrometer (c) Spectrometer test data

We measured the reflectivity of the films after IBS deposition and EB deposition, as shown in Figure S5(a). The results indicate that the multilayer films deposited by IBS exhibit a reflectivity close to 100%. After depositing the grating layer via EB deposition, the reflectivity decreased, primarily due to insufficient vacuum conditions, which caused doping of SiO or SiO_2. This also led to a reduced refractive index for the Si grating layer, which was only around 3.1.

We indirectly obtained the -1st order diffraction efficiency by measuring the 0th order reflection energy through the metasurface using PHOTON RT Spectrophotometer(Figure S5(b)), as directly measuring Littrow diffracted light is challenging. The relative diffraction efficiency for the -1st order is as follows:

$$ef_{-1} = \frac{100\% - loss - ef_0}{100\% - loss} \tag{S12}$$

where ef_{-1} is -1st order relative diffraction efficiency, ef_0 is measured 0th order efficiency and $loss$ is the film absorption.

As mentioned in the manuscript, the metasurface contains only the 0th and -1st orders, so measuring the relative efficiency using the above method is considered feasible. The spectrometer test results are shown in Figure S5(c), where the black line represents the measured 0th order reflection efficiency, and the blue line represents the calculated relative efficiency of the -1st order.

References

[1] A. Overvig and A. Alù, "Diffractive nonlocal metasurfaces," *Laser Photonics Rev.*, vol. 16, no. 8, p. 2100633, 2022, doi: 10.1002/lpor.202100633.

[2] H. Yan, X. Wu, and J. Yang, "Application of Monte Carlo method in tolerance analysis," *Procedia CIRP*, vol. 27, pp. 281–285, 2015, doi: 10.1016/j.procir.2015.04.079.
